# Duroplasty for injured cervical spinal cord with uncontrolled swelling: protocol of the DISCUS randomized controlled trial

**DOI:** 10.1186/s13063-023-07454-2

**Published:** 2023-08-07

**Authors:** Samira Saadoun, Lukas Grassner, Maurizio Belci, Jonathan Cook, Ruth Knight, Lucy Davies, Hasan Asif, Ravindran Visagan, Mathew J. Gallagher, Claudius Thomé, Peter J. Hutchinson, Argyro Zoumprouli, Julia Wade, Nicola Farrar, Marios C. Papadopoulos

**Affiliations:** 1 Academic Neurosurgery, Molecular and Clinical Sciences, St. George’s, University of London, London, UK; 2 Department of Neurosurgery, Christian Doppler Clinic, Paracelsus Medical University, Salzburg, Austria; 3 Institute of Molecular Regenerative Medicine, Spinal Cord Injury and Tissue Regeneration Center Salzburg, Paracelsus Medical University, Salzburg, Austria; 4 National Spinal Injury Centre, Stoke Mandeville Hospital, Buckinghamshire Healthcare NHS Trust, Aylesbury, Bucks UK; 5 Oxford Clinical Trials Research Unit, Botnar Research Centre, University of Oxford, Oxford, UK; 6 Liverpool Clinical Trials Centre, University of Liverpool, Liverpool, UK; 7 Surgical Intervention Trials Unit, University of Oxford, Oxford, UK; 8 Department of Neurosurgery, Innsbruck Medical University, Innsbruck, Austria; 9 Academic Neurosurgery, University of Cambridge, Cambridge, UK; 10 Neuro-Intensive Care Unit, Atkinson Morley Wing, St. George’s Hospital NHS Foundation Trust, London, UK; 11 Population Health Sciences, Bristol Medical School, University of Bristol, Bristol, UK; 12 Neurosurgery, Atkinson Morley Wing, St. George’s Hospital NHS Foundation Trust, London, UK

**Keywords:** Decompression, Dura, Duraplasty, Monitoring, Outcome, Randomized controlled trial, Spinal cord injury, Surgery

## Abstract

**Background:**

Cervical traumatic spinal cord injury is a devastating condition. Current management (bony decompression) may be inadequate as after acute severe TSCI, the swollen spinal cord may become compressed against the surrounding tough membrane, the dura. DISCUS will test the hypothesis that, after acute, severe traumatic cervical spinal cord injury, the addition of dural decompression to bony decompression improves muscle strength in the limbs at 6 months, compared with bony decompression alone.

**Methods:**

This is a prospective, phase III, multicenter, randomized controlled superiority trial. We aim to recruit 222 adults with acute, severe, traumatic cervical spinal cord injury with an American Spinal Injury Association Impairment Scale grade A, B, or C who will be randomized 1:1 to undergo bony decompression alone or bony decompression with duroplasty. Patients and outcome assessors are blinded to study arm. The primary outcome is change in the motor score at 6 months vs. admission; secondary outcomes assess function (grasp, walking, urinary + anal sphincters), quality of life, complications, need for further surgery, and mortality, at 6 months and 12 months from randomization. A subgroup of at least 50 patients (25/arm) also has observational monitoring from the injury site using a pressure probe (intraspinal pressure, spinal cord perfusion pressure) and/or microdialysis catheter (cord metabolism: tissue glucose, lactate, pyruvate, lactate to pyruvate ratio, glutamate, glycerol; cord inflammation: tissue chemokines/cytokines). Patients are recruited from the UK and internationally, with UK recruitment supported by an integrated QuinteT recruitment intervention to optimize recruitment and informed consent processes. Estimated study duration is 72 months (6 months set-up, 48 months recruitment, 12 months to complete follow-up, 6 months data analysis and reporting results).

**Discussion:**

We anticipate that the addition of duroplasty to standard of care will improve muscle strength; this has benefits for patients and carers, as well as substantial gains for health services and society including economic implications. If the addition of duroplasty to standard treatment is beneficial, it is anticipated that duroplasty will become standard of care.

**Trial registration:**

IRAS: 292031 (England, Wales, Northern Ireland) - Registration date: 24 May 2021, 296518 (Scotland), ISRCTN: 25573423 (Registration date: 2 June 2021); ClinicalTrials.gov number : NCT04936620 (Registration date: 21 June 2021); NIHR CRN 48627 (Registration date: 24 May 2021).

## Introduction

### Background and rationale {6a}

Cervical traumatic spinal cord injury (TSCI) is a devastating condition that causes partial or total limb paralysis, loss of sensation below the injury, difficulty breathing, problems maintaining blood pressure and body temperature, loss of bladder and bowel control, and loss of sexual function [1]. Patients with TSCI are vulnerable to complications including pressure ulcers, chronic pain, spasticity, joint stiffness, muscle contractures, delayed neurological deterioration from enlarging cord cyst (syrinx), pneumonia, and urosepsis.

We propose that, after acute severe TSCI, the swollen spinal cord becomes compressed against the surrounding tough membrane (dura) [2]. This suggests that bony decompression alone (current management) may be inadequate and that bony decompression + duroplasty (opening the dura longitudinally and suturing a dural patch to expand the space around the injured cord) is required. DISCUS is a randomized controlled trial aiming to investigate whether the addition of duroplasty to bony decompression improves outcome after TSCI, compared with bony decompression alone. DISCUS includes an optional mechanistic study to determine whether duroplasty reduces cord compression, improves cord perfusion, reduces cord ischemia, and reduces cord inflammation. Given the potential challenges of recruiting following emergency admission, DISCUS includes an integrated QuinteT recruitment intervention (QRI) to optimize recruitment and informed consent processes [3].

## Objectives {7}

### Primary

The primary objective is to test the hypothesis that, in patients with severe cervical TSCI, bony + dural decompression, compared with bony decompression alone, improves American Spinal Injury Association Impairment Scale (AIS) motor score (AMS) at 6 months.

### Secondary

The secondary objective is to test the hypothesis that, in patients with severe cervical TSCI, bony + dural decompression, compared with bony decompression alone, improves neurological outcome at 6 months, improves functional outcomes at 6 months, improves quality of life at 6 and 12 months, improves MRI features, increases spinal cord perfusion, reduces spinal cord inflammation at the injury site, and is safe.

## Trial design {8}

DISCUS is a randomized, controlled, multi-center trial with two trial arms: (1) surgical decompression including laminectomy (standard of care, control), and (2) surgical decompression including laminectomy + duroplasty. It is a superiority trial, i.e., aims to show that the trial arm that includes duroplasty improves outcome compared with the control arm. It has a parallel group design, i.e., participants are randomized 1:1 to receive only one of the two treatments: standard of care with laminectomy vs. standard of care with laminectomy + duroplasty. The trial is patient- and assessor-blinded, i.e., neither the patient nor the assessor of the primary outcome at 6 months know the trial treatment. We excluded thoracic TSCIs because most have severe injuries with complete paralysis without any significant spontaneous motor recovery [4, 5] and because the AMS are not sensitive to segmental recovery in the thoracic region.

### Optional mechanistic study

A subset of patients, at least *N* = 50 (at least 25/arm), will undergo monitoring from the injury site of intraspinal pressure (ISP) + spinal cord perfusion pressure (SCPP) and/or microdialysis (MD), for up to 5 days after surgery.

### QuinteT recruitment intervention (QRI)

This is to optimize recruitment and informed consent and is embedded during the first 2 years of recruitment (UK centers only).

## Methods: participants, interventions, and outcomes

### Study setting {9}

In the UK, there are two types of participating centers: (1) recruiting centers (major trauma centers), where surgery and post-operative care take place, and (2) rehabilitation centers (spinal injury units), where the participants are transferred, once the acute care has finished. For the 6-month assessments, most patients are expected to be at the spinal injuries units. DISCUS started as a UK study but has now expanded to international centers. UK and international centers use the same protocol of patient assessment, follow-up, investigations, outcomes, etc., and randomize via the same process using RedCap. The currently registered centers are listed at the DISCUS trial website [6].

### Eligibility criteria {10}

#### Inclusion

Patients will be eligible for inclusion in the trial if they meet all of the following criteria: (1) age ≥ 16 years, (2) severe cervical (C2–T1) TSCI (AIS grade A–C), (3) deemed to require and be suitable for surgery that includes laminectomy by local surgeon, (4) surgery within 72 h of TSCI, and (4) able to provide informed consent (UK allows proxy consent).

#### Exclusion

Patients will be excluded from participating in the trial if they meet any of the following criteria: (1) probable dural tear due to TSCI, (2) life-limiting or rehabilitation-restricting co-morbidities, (3) thoracic or lumbar TSCI, and (4) other central nervous system diseases.

### Who will take informed consent? {26a}

The local principal investigator (P.I.) retains overall responsibility for obtaining consent from participants at their site. Anyone delegated responsibility to obtain consent, typically a neurosurgeon, must be duly authorized, trained, and competent according to the protocol, principles of Good Clinical Practice (GCP) and Declaration of Helsinki. The operating surgeon does not have to be GCP-trained. Informed consent must be obtained before the participant undergoes trial procedures and before randomization. At least 2 h are allowed between explaining the study; that includes providing the patient information sheets, and obtaining consent, to enable the person giving consent to consider the information provided. Participants are free to withdraw at any time from the trial without giving reasons and without prejudicing further treatment. All participants will continue to be followed up as per routine standard of care. Withdrawn participants will be asked whether the data acquired up to the point of withdrawal can be retained. The reason for withdrawal is recorded in the case report form (CRF). Wherever possible, informed consent is obtained from the patient. If the patient verbally consents to participate, but is unable to sign because of hand weakness as a result of the TSCI, then the form is signed by an independent healthcare professional as a witness. Some patients may have impaired consciousness from sedative drugs. In these cases, DISCUS allows proxy consent, depending on the rules and regulations of the country. Patients who enter DISCUS via proxy consent and who regain capacity in hospital are informed about the trial and retrospective consent to continue participation is sought from them. The consent form is approved by the ethics committee of each participating country and follows GCP, local regulatory, and legal requirements.

### Additional consent provisions for collection and use of participant data and biological specimens {26b}

#### Consent for monitoring from injury site

The signed consent form will require the consenting person to indicate whether the patient has consented only for the clinical study or for both the clinical study plus optional injury site monitoring study. MD specimens may be analyzed on site for standard metabolites. Leftover MD samples are stored on site and periodically shipped (anonymously) to St. George’s Hospital for further assay of cytokines. MD samples are not human tissue as they are fluid collected through a 20-kDa dialysis membrane.

#### Consent for QRI (UK only)

Written consent may be sought from patients/their legal representative to audio-record the recruitment discussion and/or take part in an interview with a QRI researcher. Patients may consent to the audio recording and/or interview and decline participation in DISCUS or vice versa, decline the audio recording and/or interview but consent to participation in DISCUS. A separate form will record consent to take part in QRI.

## Interventions

### Explanation for the choice of comparators {6b}

#### Control arm

All patients undergo surgery that involves bony decompression ± spinal fixation. The surgical approach (anterior vs. posterior fixation, number of levels) is at the discretion of the local clinicians as is the management of physiological parameters (blood pressure, arterial CO_2_, O_2_, etc.). The study requires that all patients have laminectomy spanning the levels of swollen cord based on the MRI, which requires a posterior approach.

#### Intervention arm

Patients randomized to the duroplasty arm will also undergo expansion duroplasty, dorsally, under the same general anesthetic. Therefore, patients undergo either a posterior approach only or combined anterior + posterior approach.

#### Monitoring probes

The patients for the optional mechanistic studies, in the control arm or intervention arm, have a pressure probe and/or a MD catheter inserted intradurally posteriorly at the site of injury for up to 5 days and removed in ICU.

### Intervention description {11a}

#### Duroplasty

Though neurosurgeons at participating centers are familiar with duroplasty, the technicalities of the procedure are discussed at the initial site visit and in online training videos. The length of the duroplasty spans the length of the swollen cord estimated from the preoperative MRI. Duroplasty is performed by suturing an elliptical patch of artificial dura about 1 cm longer than the dural incision and about 2 cm wide to the dural edge [7]. We recommend the following: (1) a wound drain be placed on gravity, (2) the skin be sutured and covered with waterproof adhesive iodine-impregnated dressing to minimize the risk of wound infection and of CSF leak, (3) the wound drain and dressing be removed at 1 week and the sutures at 2 weeks, and (4) CSF leak be treated by placing extra sutures ± lumbar drain.

#### Monitoring probes

Probe insertion is done during the surgery to fix the spine. Each probe is tunneled through the skin and soft tissue, inserted intradurally with the tip positioned at the site of maximum swelling [8, 9]. The technicalities of probe insertion, tunneling, suturing to the skin, and removal are discussed at the initial site visit and in online training videos.

### Criteria for discontinuing or modifying allocated interventions {11b}

The local PI may discontinue a participant from the study, at any time if (1) following consent, but prior to surgery, a patient clinically deteriorates such that surgery is no longer considered in the patient’s best interests; (2) following consent, but prior to surgery, surgery is postponed beyond the 72-h window; or (3) during surgery, the surgeon considers use of duroplasty is not appropriate or possible; (4) during surgery, where the surgeon discovers that the dura is substantially damaged by the penetrating injury; and (5) a patient declines to continue participation in the trial. Where possible, participants who have withdrawn from the trial intervention should continue to be followed up, unless they withdraw consent for this.

### Strategies to improve adherence to interventions {11c}

We anticipate that surgeons will adhere to the procedure allocated by randomization.

### Relevant concomitant care permitted or prohibited during the trial {11d}

DISCUS was designed to avoid controversies in the medical, anesthetic, and surgical management of TSCI patients (e.g., target blood pressure, type of anesthetic agent, timing of surgery) [10] by allowing centers to time the surgery and medically manage patients according to local clinical practices. Any influence of variations in the timing of surgery and in the medical management on outcome measures are expected to be balanced between the two trial arms of DISCUS given the randomized nature of the study.

### Provisions for post-trial care {30}

Patients will be managed as per standard of care. The sponsors, St. George’s, University of London (UK) and University of Salzburg (non-UK), hold indemnity insurances to cover participants for injury caused by their participation in the clinical trial.

### Outcomes {12}

#### Primary (motor)

The primary outcome (motor) is as follows: change in AIS motor score at 6 months vs. baseline. We use the 2019 AIS form, which is a standardized, validated, clinician-administered scale to classify severity of TSCI [11]. For intubated patients, sedation is lightened sufficiently to allow assessment at the discretion of local doctors. Participants have the AIS done on admission before randomization and before surgery (by neurosurgeon or physiotherapist) and at 6 months (by physiotherapist, rehabilitation doctor, or neurosurgeon). AIS motor scores range from 0 to 100 with higher scores indicating better outcomes.

#### Secondary (neurology)

The secondary outcome (neurology) is as follows: change at 6 months vs. baseline in (1) light touch score (range 0 to 112, higher scores indicate better outcomes), (2) Pin prick score (range 0 to 112, higher scores indicate better outcomes), and (3) AIS grades 1 and 2 are part of the AIS. From the AIS chart, we derive the AIS grade (A—motor and sensory complete impairment, B, C, D, E—normal).

#### Secondary (function)

The secondary outcome (function) at 6 months is as follows: (4) CUE-Q (Capabilities of Upper Extremity Questionnaire) [12]—range 0 to 128 with higher scores indicating better function, (5) dynamometer (hand grip strength) measured in kg, (6) WISCI-II (Walking Index for Spinal Cord Injury) [13]—range 0 to 20 with higher scores indicating better function, (7) SCIM-III (Spinal Cord Injury Independence Measure)—range 0 to 100 with higher scores indicating better function [14]. We use these scales, which are established in TSCI, to test if motor improvements are associated with improvements in function including urinary and anal sphincters.

#### Secondary (quality of life)

The secondary outcome (quality of life) is as follows: (8) SF-36 (Short Form 36) [15] at 6 and 12 months. We aim to test if functional improvements translate to improvements in quality of life.

#### Secondary (safety)

The secondary outcome (safety) is as follows: (9) number of re-operations on the spine within 1 year, (10) total length of hospital stay (days), (11) surgical complications and adverse events, (12) overall survival—from randomization to death or end of follow-up (1 year), (13) MRI 2 weeks: size of pseudomeningocele (cm^3^), (14) MRI 6 months: spinal deformity (change in Cobb angle at 6 months versus baseline); size of pseudomeningocele (cm^3^) ((9)–(13) are objective measures). (14) (spinal deformity) is a standardized tool. Safety of duroplasty is assessed not only by comparing complications + adverse events but also by comparing the number of re-operations on spine, total length of hospital stay, overall survival, and spinal deformity in the duroplasty vs. non-duroplasty arms.

#### Secondary (mechanisms)

The secondary outcome (mechanisms) is as follows: (15) MRI at 2 weeks (all patients): length of cord compression (mm), (16) MRI at 6 months (all patients): length of cord tether (mm), length of cord syrinx (mm), (17) pressure monitoring at injury site (mechanistic study patients): mean daily ISP, mean daily SCPP, (18) MD monitoring at injury site (mechanistic study patients): mean daily metabolites (glucose, lactate, pyruvate, lactate to pyruvate ration —LPR, glutamate, glycerol). Mean daily cytokines. (15) and (16) will be measured on MRI by two independent experts. (17) and (18) are objective measures independent of assessor. The aim is to determine the mechanism by which duroplasty improves outcome. We will investigate if dural + bony decompression more effectively decompresses the swollen cord than bony decompression alone (lower ISP, higher SCPP, CSF around injured cord on MRI). More effective decompression by duroplasty is predicted to improve cord metabolism, compared with bony decompression alone (higher glucose, lower LPR, lower glutamate, lower glycerol) and reduce cord inflammation (lower pro-inflammatory and higher anti-inflammatory cytokines). On the 6-month MRI, the extra space created by duroplasty is predicted to reduce cord tethering and syrinx.

### Participant timeline {13}

The SPIRIT figure summarizes the participant timeline.

SPIRIT figure. Schedule of enrollment, interventions, and assessments



### Sample size {14}

Assuming a 6-month improvement in change in AIS motor score (ΔAMS) from a mean (SD) of 17 (25) in the control arm to 28 (25) in the intervention arm, allowing for 15% patient loss to follow-up by 6 months (including patient death which is estimated to be around 2–3% by 6 months), a total sample size of 222 patients is required with statistical power of 85% and 2-sided significance level of 5%. The assumed difference in ΔAMS and SD are based on our exploratory study [7] and are supported by data from the European Multicenter Study about Spinal Cord Injury (EMSCI) database and other published series [16, 17] in which ΔAMS is between 15 and 18 with SD ranging from 15 to 25. While recruitment is in progress, the statistical power will be revised from 85 to 90% if it becomes clear that the new recruitment figure (*N* = 260) is achievable. The final decision to increase recruitment will be made by the TSC.

#### Mechanistic study

Since the proposed mechanistic studies are intended to be hypothesis-generating, no formal sample size calculation has been performed.

### Recruitment {15}

DISCUS may face challenges to successful recruitment and consent processes because the pool of eligible patients is relatively small (9–13 patients per year per center), recruitment is led by busy neurosurgical trainees who may not prioritize trial recruitment in high-pressure surgical environments, consent may be by proxy at a stressful time for family members, and there may be issues with equipoise if surgeons favor an anterior approach and if patients or families favor the novel procedure (bony decompression + duroplasty) over standard procedures. Given these challenges, we have integrated a QRI [3] during the first 15 months of recruitment to the main trial for UK centers. QRI is a two-stage intervention to identify then address recruitment and consent challenges: Phase 1: understanding recruitment, involves collection and analysis of basic screening log data (numbers screened, eligible, approached and recruited to the trial) [18], alongside collection and analysis of qualitative data (healthcare professional and/or participant interviews and/or recruitment discussions). Findings are triangulated to develop hypotheses regarding recruitment and informed consent processes [3, 19]. Phase 2: Feedback to the CI/TMG and TSC and plan of action. In practice, these two phases will run in tandem, with QRI findings fed back iteratively to the CI/TMG to agree appropriate actions.

## Assignment of interventions: allocation

### Sequence generation {16a}

Participants are randomly allocated 1:1 to the treatment options. Randomization allocation is implemented using a minimization algorithm which accounts for the following factors: age group (< 40, 40–60, > 60 years) and country (including England, Wales, Scotland, Northern Ireland, and each European country).

### Concealment mechanism {16b}

The minimization algorithm is seeded with a number of allocations and a non-deterministic probabilistic element is introduced to prevent predictability of the treatment allocation. To ensure concealment, we use a validated password RedCap website and ensure that the web-based secure system (RRAMP) will not release the randomization code until the patient has been recruited into the trial.

### Implementation {16c}

Participants are enrolled by the local P.I. and/or co-P.I. Randomization is done using a RRAMP provided by the Oxford Clinical Trials Research Unit. The randomization result is then emailed to the local P.I. and co-P.I. who assign the participants to the allocated intervention.

## Assignment of interventions: blinding

### Who will be blinded {17a}

The patients and the assessors of primary and secondary (grip strength, CU-Q, WISCI II, SCIM III, SF-36, MRI) outcomes are blinded to trial treatment arm. Baseline AMS is assessed at presentation, prior to randomization. When randomized, the patient is not informed which trial arm they have been allocated to. The 6 months post-randomization assessment is performed by a physiotherapist, rehabilitation doctor, or neurosurgeon who is unaware of the trial arm. In the unlikely event that the assessor becomes accidentally unblinded, the data will still be used. The study will report the number of unblinded assessments that have been performed.

### Procedure for unblinding if needed {17b}

We do not foresee a situation in which a DISCUS patient will require emergency unblinding.

## Data collection and management

### Plans for assessment and collection of outcomes {18a}

Patient characteristics are transferred from hospital notes to the trial database. AIS is a standard paper chart in the patient notes transferred to the trial database. MRI and CT, normally stored electronically at each hospital, are electronically transferred for further analysis. Length of hospital stay and number of re-operations on spine are obtained from hospital records and/or the patient. SF-36 is completed online, on paper or by phone and transferred to the trial database. CUE-Q, WISCI-II, and SCIM-III need patient exam; the results are in the patient records and are transferred to the trial database. Grip strength is assessed by a dynamometer. Complications and adverse events are recorded in patient notes and reported by local investigators to the trial database. Mortality is obtained from hospital records or reported by local investigators or the family doctor.

#### Mechanistic study

ISP, MAP, and MD measurements of metabolites will be recorded locally by the ICU nurses on patients’ charts and entered onto the trial database. MD measurements of cytokines will also be recorded anonymously and added to the trial database.

#### QRI data

Audio recordings and transcripts are held on University of Bristol encrypted drives for a maximum of 10 years after the study ends.

### Plans to promote participant retention and complete follow-up {18b}

In the UK, outcome measures are collected at the rehabilitation centers. Participants are either still inpatients at 6 months or are recalled for routine outpatient appointments that include completing the outcomes. Non-UK centers typically collect the outcome measures during outpatient appointments. Form completion is monitored by the DISCUS trial manager. In case of incomplete eCRFs, the local P.I. and/or co-P.I. will be contacted.

### Data management {19}

Trial data are captured on the DISCUS trial database using REDCap, either directly entered or transferred from hospital notes. Trial data are collected on trial specific documents, e.g., questionnaires and CRFs. The data are processed in accordance with data protection rules. Access is granted to authorized representatives from the sponsor, host institution, and regulatory authorities to permit trial-related monitoring and inspections.

### Confidentiality {27}

All trial-specific documents, except for the signed consent form and follow-up contact details, refer to the participant with a unique study participant number/code and not by name. The participants are identified by a participant identification number ± year of birth on all study documents and any electronic database. Participant identifiable data are stored separately from study data. All trial data are stored securely in offices only accessible by swipe card by the central coordinating team staff in Oxford and authorized personnel. QRI data will be held securely on a University of Bristol encrypted server, with restricted access for authorized personnel, for a maximum of 10 years after the study ends.

## Plans for collection, laboratory evaluation, and storage of biological specimens for genetic or molecular analysis in this trial/future use {33}

### Onsite analysis of MD samples

MD is widely used for the management of patients with traumatic brain injury in ICU. The MD fluid is collected in standard vials hourly and periodically analyzed in a bedside MD analyzer for glucose, lactate, pyruvate, glutamate, and glycerol.

### Analysis of cytokines from MD samples

Leftover MD samples are stored locally in a freezer and periodically shipped to St. George’s for multiplex ELISA of cytokines (GROα, IL1α, IL1b, IL4, IL8, IL10, IP10, MCP1, MIP1α, MIP1β). These metabolites and cytokines are stable with freeze-thaw, for several hours at 25 °C and indefinitely at − 20 °C; thus, measurements are independent of variability in sample collection [20]. The stored vials are anonymized for analysis and destroyed once the measurements have been made.

### Staff training

ICU staff are trained not to intervene to treat ISP, SCPP, and MD values; otherwise, those patients in the mechanistic study may receive different medical management from those not in the mechanistic study.

## Statistical methods

### Statistical methods for primary and secondary outcomes {20a}

#### Primary outcome

The primary analysis will be performed according to the intention-to-treat principle and will be analyzed at a 2-sided 5% significance level. The primary outcome, ΔAMS from baseline to 6 months, will be analyzed using a generalized estimating equation (GEE) or mixed model for repeating measures (MMRM) model (depending on the distribution of the primary outcome), adjusted for randomized treatment and baseline AIS grade. Minimization factors (age group, country) will be adjusted for as fixed effects. A secondary analysis will be performed adjusting for other important, pre-specified prognostic factors.

#### Secondary outcomes

MMRM will be used to evaluate difference over time by treatment group. MMRM models will be adjusted for minimization factors, treatment, baseline score, visit, and a treatment by visit interaction as fixed effects, with patient included as a random effect. Differences between treatment groups will be reported as mean differences with 95% confidence intervals. Binary outcomes will be assessed using chi-squared tests and logistic regression analyses, adjusting for baseline and minimization factors. Normality will be assessed for continuous outcome measures, and differences between treatment arms will be assessed using a *t*-test or Mann-Whitney *U* test and presented as mean differences with 95% confidence intervals or median differences with interquartile range. Linear regression or GEE models will be used to adjust for baseline, minimization, and other important prognostic factors. Survival analysis methods will also be used to compare differences between the treatment groups for overall survival. Time to overall survival will be displayed using Kaplan-Meier plots. Absolute (death rates at 12 months) and relative differences (hazard ratios) will be reported together with 95% confidence intervals.

### Interim analyses {21b}

No formal interim analyses are anticipated prior to completion of follow-up for the designated time points. The data safety and monitoring committee (DSMC) may request interim analyses at any point in the trial, which will be performed by the trial statistician.

## Methods for additional analyses (e.g., subgroup analyses) {20b}

### Mechanistic studies

We will plot, for each of the two trial arms, mean ± standard deviation daily values for ISP, SCPP, tissue glucose, lactate, pyruvate, LPR, glutamate, glycerol, GROα, IL1α, IL1b, IL4, IL8, IL10, IP10, MCP1, MIP1α, and MIP1β.

### QuinteT recruitment intervention

Simple descriptive data on numbers screened, eligible, approached for a discussion about, and recruited to the trial will be compared across UK sites to identify points on the recruitment pathway where patients are being “lost” to recruitment. Screening log findings for individual sites will be triangulated with findings from qualitative data, particularly interviews with site team members, to identify barriers and potential facilitators and inform plans for optimizing recruitment and informed consent [3].

## Methods in analysis to handle protocol non-adherence and any statistical methods to handle missing data {20c}

AIS assessment involves systematically completing a form, and, therefore, we do not anticipate missing values. Any missing date for the primary outcome (AMS) will be reviewed on a case-by-case basis by an independent clinician who is blinded to the treatment the patient received. Forms for secondary outcomes that are returned partially complete will be analyzed according to the scoring instructions for each questionnaire.

## Plans to give access to the full protocol, participant-level data, and statistical code {31c}

The DISCUS protocol is available online from the NIHR and the DISCUS websites. After closure of the trial and data analyses, the data generated will remain the responsibility of St. George’s, University of London (“the contractor”). All requests for data should be directed to the contractor and managed by the contractor, in accordance with the St. George’s data sharing policies. Release of data will be subject to a data use agreement between the contractor and the third party requesting the data.

## Oversight and monitoring

### Composition of the coordinating center and trial steering committee {5d}

The trial management group (TMG) consists of personnel involved in daily operational issues in the management of the trial, is chaired by the trial manager, and acts upon advice/recommendations received by the sponsor and/or the DSMC. The trial steering committee (TSC) consists of personnel directly involved in the trial and independent individuals. The TSC concentrates on the progress of the trial in relation to protocol compliance and review of any participant safety considerations including the review of any recommendations made by the DSMC if relevant to the trial and to advise the sponsor and/or CI and Co-CI of any decisions.

### Composition of the data monitoring committee, its role and reporting structure {21A}

The DSMC is a committee independent of the sponsor, composed of two external neurosurgeons and an external statistician, established to assess at intervals the progress of the clinical trial, the safety data, and the critical efficacy endpoints and to recommend to the TSC whether to continue, modify, or stop the trial. The DSMC members have no competing interests. A DSMC charter is in place.

### Adverse event reporting and harms {22}

The following are reported by sites as serious adverse events (SAEs) to the central team within 24 h, only if deemed potentially directly related to duroplasty or probes: (1) death, (2) meningitis, (3) redo spinal surgery, (4) worsening of AIS grade, (5) wound infection that requires antibiotics, and (6) wound breakdown that requires surgical debridement. The following complications are collected but do not require SAE reporting: (1) death related to reasons other than duroplasty or probes, (2) repair of CSF leak not requiring general anesthetic, (3) redo spinal surgery unrelated to duroplasty or probes, (4) worsening of AIS grade (if deemed not related to duroplasty or probes, this does not need reporting and is picked up in 6-month AIS assessment), (5) deep wound infection that needs antibiotics if deemed not related to duroplasty or probes, (6) wound breakdown that requires surgical debridement if deemed not related to duroplasty or probes, (7) pseudomeningocele, (8) pressure ulcers during 1st week post op., (9) readmissions to hospital. Events collected as complications from sites will be reviewed by the TMG and assessed by clinicians unrelated to surgery. DSMC and TSC will get a list of SAEs. The DISCUS Trial Office is responsible for reporting SAEs, where appropriate, to the sponsor and the research ethics committee (REC) within required timelines.

### Frequency and plans for auditing trial conduct {23}

A risk assessment was undertaken, and a proportionate monitoring plan has been put in place to decide on the extent and nature of any on-site monitoring. Central monitoring of incoming data and operational aspects of the trial are done by the Oxford Surgical and Interventional Trials Unit (SITU). An independent DSMC has been established with an independent chair and suitable multi-disciplinary representation. The DSMC meets at least annually. Triggered monitoring is performed by SITU as determined by risk assessment and monitoring plan following GCP principles. Data are evaluated for compliance with the protocol and accuracy in relation to source documents.

### Plans for communicating important protocol amendments to relevant parties (e.g., trial participants, ethical committees) {25}

St. George’s, University of London may make a non-substantial amendment at any time during the trial. If St. George’s, University of London wishes to make a substantial amendment to the research ethics committee application or the supporting documents, St. George’s, University of London must submit a valid notice of amendment to the research ethics committee for consideration. It is St. George’s, University of London’s responsibility to decide whether an amendment is substantial or non-substantial for the purposes of submission to the research ethics committee. Amendments need to be notified to the UK Health Research Authority and communicated to the research office and local research team at each participating organization.

## Dissemination plans {31a}

Following the conclusion of patient recruitment and follow-up and data analysis, the chief investigators shall liaise with all investigators to consolidate data and results and submit a manuscript for peer-review with a view to publish in a reputable academic journal. Results will be disseminated to spinal cord injury patients via the UK Spinal Injuries Association and Wings for Life.

## Discussion

Several lines of evidence suggest potential benefits of duroplasty: (1) *Exploratory studies*. One study assessed safety and the effect on intraspinal pressure (ISP) and spinal cord perfusion pressure (SCPP) of duroplasty after TSCI [7]. Duroplasty was found to be safe: 50% patients had non-compressive pseudo-meningocele that disappeared at 6 months, with no wound infection, no persistent cerebrospinal fluid (CSF) leak, and no worsening neurology. Compared with bony decompression, bony + dural decompression reduced ISP by about 10 mmHg and increased SCPP by about 15 mmHg. Publications of TSCI patients treated with bony decompression + duroplasty report generally favorable outcomes and no complications [21–25]. Duroplasty is commonly performed for Chiari I malformation, with ample evidence of safety [26]. (2) *ISP monitoring*. A key finding of the Injured Cord Pressure Evaluation study is that ISP remains high with low SCPP even after anterior + posterior bony decompression; this suggests that the dura contributes to cord compression [8, 27]. (3) *Evidence from MRIs*. In a cohort of 65 TSCI patients without bony compression, dural compression was evident on MRI as lack of cerebrospinal fluid (CSF) around the injured cord [28]. The extent of dural cord compression increased with increasing severity of TSCI and resolved slowly (*t*_1/2_ = 9 days). (4) *Animal studies*. Reducing ISP by duroplasty or genetic manipulation to limit cord swelling improved outcome in numerous rodent TSCI models [22, 29–33]. These studies show that, after TSCI, the cord swells against the dura from cord hematoma and edema. In rats, duroplasty has beneficial effects other than lowering ISP, including less cord inflammation, less cord scarring, and smaller syrinx. The studies suggest that fibrin sealants may cause cord compression, and we have, therefore, avoided their use in our duroplasty trial. (5) *Analogy with traumatic brain injury*. The dura is unstretchable [34]. It is established that the dura compresses swollen brain [35]; thus, decompression for traumatic brain injury is bony + dural decompression, shown to lower mortality in the RESCUEicp randomized controlled trial [36].

For these reasons, neurosurgery centers worldwide are currently performing duroplasty in TSCI patients without robust randomized evidence [21–25]; DISCUS is the first prospective trial to address this question.

## Trial status

This paper is based on DISCUS Study Protocol version 6.0, dated 30 May 2022. Recruitment started on 9 October 2022 and is currently estimated to continue for 4 years.

## Data Availability

The chief investigators, Professor Marios C. Papadopoulos and Dr Samira Saadoun, are the custodians of the data. The DISCUS database is completely in the hand of the Oxford Surgical and Interventional Trials Unit (SITU) and therefore separated from the investigators and from the participating sites. During the trial, access to the trial data will be limited to the monitoring and data cleaning activities required to deliver the trial objectives and for the purposes of regulatory and safety reviews. After database closure, the responsible statistician will provide the calculations (details of the analyses will be determined in the statistical analysis plan) without interaction with the chief or principal investigators. DISCUS trial data will be accessible to sponsor representatives, representatives of the Surgical Intervention Trials Unit and the Oxford Clinical Trials Research Unit at the University of Oxford, and responsible individuals identified by the sponsor, including representatives of regulatory authorities and NHS trust. The DISCUS trial data is stored on the trial database and any paper documents are stored in a locked filing cabinet at the trial office. The final dataset will be archived electronically on a University of Oxford Server. Participating sites will store their site and patient files in accordance with the requirements stipulated by their local governance policies. Any data required to support the protocol can be supplied on request.

## Abbreviations

AIS: American Spinal Injury Association Impairment Scale
AMS: AIS motor score
CI: Chief investigator
CRF: Case report form
CSF: Cerebrospinal fluid
CUE-Q: Capabilities of Upper Extremity Questionnaire
ΔAMS: Change in AIS motor score
DISCUS: Duroplasty for injured cervical spinal cord with uncontrolled swelling
DSC: Data safety committee
EMSCI: European Multicenter Study about Spinal Cord Injury
GCP: Good Clinical Practice
GEE: Generalized estimating equations
GROα: Growth-related oncogene alpha
ICU: Intensive care unit
IL10: Interleukin 10
IL1α: Interleukin 1 alpha
IL1β: Interleukin 1 beta
IL4: Interleukin 4
IL8: Interleukin 8
IP10: Interferon gamma-induced protein 10
ISP: Intraspinal pressure
LPR: Lactate to pyruvate ratio
MCP1: Monocyte chemoattractant protein 1
MD: Microdialysis
MIP1α: Macrophage inflammatory protein 1 alpha
MIP1β: Macrophage inflammatory protein 1 beta
MMRM: Mixed models for repeated measures
MRI: Magnetic resonance imaging
NIHR: National Institute for Health Research (UK)
PI: Principal investigator
QRI: QuinteT recruitment intervention
SAE: Serious adverse event
SCIM-III: Spinal Cord Independence Measure version III
SCPP: Spinal cord perfusion pressure
SF-36: Short Form 36
SITU: Surgical and Interventional Trials Unit
TMG: Trial management group
TSC: Trial steering committee
TSCI: Traumatic spinal cord injury
WISCI-II: Walking Index for Spinal Cord Injury version II
